# Supplementary material for: Oxygen diffusion in ellipsoidal tumour spheroids
Source: J R Soc Interface. 2018 Aug 15;15(145):20180256. doi: 10.1098/rsif.2018.0256 (PMC6127169; doi:10.1098/rsif.2018.0256)
Supplement: S1 Appendix. Mathematical appendix and full derivations. [file rsif20180256supp1.pdf]

Oxygen diffusion in ellipsoidal tumor spheroids - S1 Supplementary mathematical appendices

David Robert Grimes[1], Frederick J. Currell [1],

1 Centre for Advanced and Interdisciplinary Radiation Research (CAIRR) School of Mathematics and Physics, Queen's University Belfast, BT7 1NN, Northern Ireland, UK.

* d.r.grimes@qub.ac.uk

Abstract

S1: Oxygen diffusion in ellipsoidal tumor spheroids - Mathematical appendices.

Ellipsoidal oxygen tension derivations

Prolate spheroids

In a prolate spherical geometry, we employ a prolate spherical coordinate system where curves of constant σ are prolate spheroids, whilst curves of constant τ correspond to hyperboloids of revolution [1]. One can imagine this co-ordinate system as a series of concentric ellipses, akin to how one would envision the spherical co-ordinate system as concentric spheres. The parameter σ defines an ellipsoidal surface in this co-ordinate system. Under azimuthal symmetry, the Laplacian in these coordinates is given by

$$\nabla^2 P = \frac{1}{f^2(\sigma^2 - \tau^2)} \left\{ \frac{\partial}{\partial \sigma} \left[\left(\sigma^2 - 1\right) \frac{\partial P}{\partial \sigma} \right] + \frac{\partial}{\partial \tau} \left[(1 - \tau^2) \frac{\partial P}{\partial \tau} \right] \right\} \tag{1}$$

where f is the distance between the ellipse focal length and centre. We need to solve a reaction-diffusion equation for oxygen partial pressure P, subject to appropriate boundary conditions. This is analogous to the spherical case, where oxygen diffusion occurs so rapidly we work under a steady-state assumption ($\frac{\partial P}{\partial t} = 0$) [2,3]. Under this schema, oxygen diffuses through tissue, consumed at a rate $a\Omega$, and the reaction diffusion equation to be solved can be re-written as $\nabla^2 P = \frac{a\Omega}{D}$, where D is the oxygen diffusion constant in water. Letting $\frac{a\Omega}{D} = k$, the resultant nonhomogenous partial differential equation can be solved by separation of variables. Formally the solution should be a summation over an infinite series of products of functions, in τ and σ. For brevity we only consider one such pair of functions and note that all other terms in the summation are zero due to the boundary conditions. Hence we write $P(\tau, \sigma)$ as a separable pair of functions $P = F(\tau)G(\sigma)$. One can very directly proceed to the solution by noticing that the boundary conditions on the outer surface of the spheroid implies that $F(\tau)$ must be a constant. Hence direct substitution of $P = G(\sigma)$ into equation 1 allows us to directly proceed to the solution given in 10. Alternatively one can proceed more formally as we do below. Rearranging 1 and substituting for k gives

$$f^2(\sigma^2 - \tau^2)k = \frac{\partial}{\partial \sigma} \left((\sigma^2 - 1) \frac{\partial P}{\partial \sigma} \right) + \frac{\partial}{\partial \tau} \left((1 - \tau^2) \frac{\partial P}{\partial \tau} \right). \tag{2}$$

Extracting the homogeneous parts in τ and σ and using the usual constant of separation gives

$$\frac{\partial}{\partial\tau}\left((1-\tau^2)\frac{\partial F}{\partial\tau}\right)-\lambda F=0, \tag{3}$$

$$\frac{\partial}{\partial\sigma}\left((\sigma^2-1)\frac{\partial G}{\partial\sigma}\right)+\lambda G=0. \tag{4}$$

Equation 3 has the general solution

$$c_1\mathbb{P}_{\frac{1}{2}\left(-1+\sqrt{1+4\lambda}\right)}(\tau)+c_2\mathbb{Q}_{\frac{1}{2}\left(-1+\sqrt{1+4\lambda}\right)}(\tau). \tag{5}$$

where $\mathbb{P}$ and $\mathbb{Q}$ are notations for associated Legendre functions of the first and second kind respectively. $\mathbb{Q}_{\frac{1}{2}\left(-1+\sqrt{1+4\lambda}\right)}(\tau)$ gives rise to unphysical behaviours and can be rejected, i.e. $c_2=0$. Furthermore, boundary conditions require the Legendre polynomial $\mathbb{P}_{\frac{1}{2}\left(-1+\sqrt{1+4\lambda}\right)}(\tau)$ to have an index of 0, i.e.

$$\frac{1}{2}\left(-1+\sqrt{1+4\lambda}\right)=0, \tag{6}$$

or $\lambda=0$. Hence the solution takes the form $\mathbb{P}=P_0(\tau)G(\sigma)$ with the constant c_1 being absorbed into $G(\sigma)$. Substituting this into 2 then gives (using primes to denote differentiation with respect to σ)

$$f^2(\sigma^2-\tau^2)k=2\sigma G'P_0-G''P_0+\lambda GP_0. \tag{7}$$

We exploit $\int_{-1}^{1}P_0f^2(\sigma^2-\tau^2)kd\tau=\int_{-1}^{1}P_0(2\sigma G'P_0-G''P_0+\lambda GP_0)d\tau$ and explicitly making use of $P_0=1$, we arrive at

$$kf^2(\sigma^2-1/3)=2\sigma G'-G''+\lambda G, \tag{8}$$

which yields a general solution of the form

$$P=\frac{kf^2}{6}\left[\left(\sigma^2+\log(\sigma^2-1)+\frac{C_1}{2}\log\left(\frac{1-\sigma}{1+\sigma}\right)\right)-\left(\tau^2+\log(\tau^2-1)+\frac{C_1}{2}\log\left(\frac{1-\tau}{1+\tau}\right)\right)\right]+C_3, \tag{9}$$

where C_1, C_2 and C_3 are constants. To find the specific solution, we need to apply appropriate boundary conditions. On the innermost elliptical surface, σ_n, there is a no-flux condition so that $\frac{\partial P}{\partial\sigma}=0$ on this surface. Applying this to equation 9, we can ascertain that $C_1=-2\sigma_n^3$. We can further state that on the outer boundary σ_o, $P=p_o$ and on the inner surface σ_n, $P(\sigma_n)=0$. When this is solved for C_3, all τ terms cancel, leaving an expression entirely in terms of σ for the oxygen partial pressure on confocal

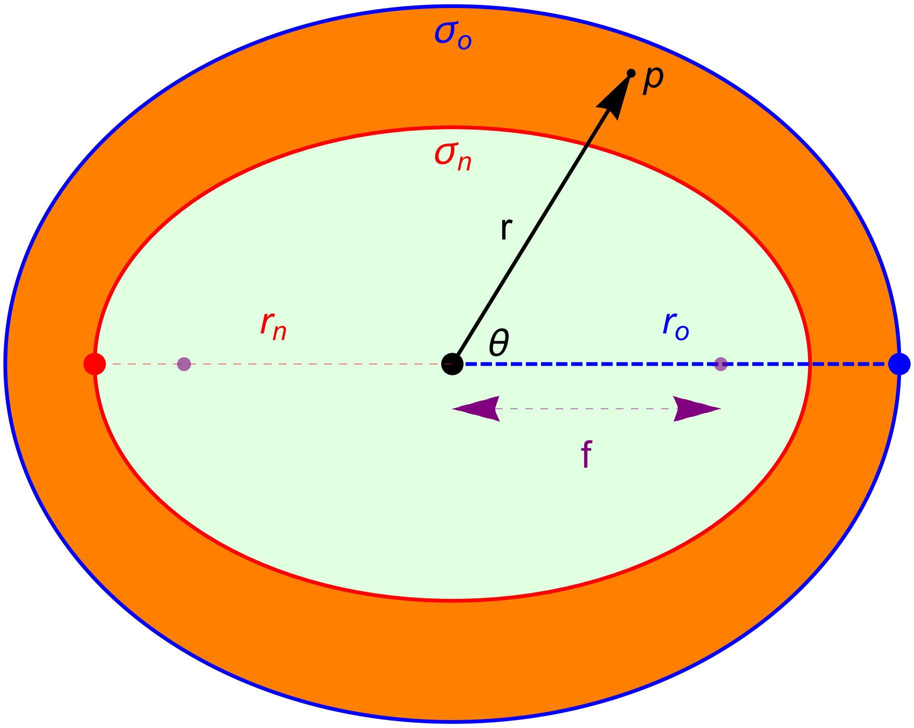

Fig 1. Re-production of geometry figure from main text

prolate elliptical surfaces of

$$P_P(\sigma) = \frac{a\Omega f^2}{6D}\left(\sigma^2 - \sigma_n^2 + \log\left(\frac{\sigma^2 - 1}{\sigma_n^2 - 1}\right) + \sigma_n^3 \log\left(\frac{(1+\sigma)(1-\sigma_n)}{(1-\sigma)(1+\sigma_n)}\right)\right). \tag{10}$$

It can be readily shown that this form satisfies the boundary conditions, and is the solution to the desired steady state reaction-diffusion equation ($\nabla^2 P = \frac{a\Omega}{D}$). To arrive at the more intuitive form shown in the main text, we need to convert from this co-ordinate system to spherical co-ordinates. Consider the geometry figure from the main text, re-produced above in figure 1. A point p on the ellipsoid is a distance r and angle θ from the centroid. It lies on some confocal ellipse with semi-major axis r_e. We know from the confocality condition that this ellipse will also have focal length f. The parametric form for the point p is $(r_e \cos\theta, \sqrt{r_e^2 - f^2}\sin\theta)$. From trigonometric arguments, we can write

$$r^2 = r_e^2 \cos^2\theta + r_e^2 \sin^2\theta - f^2 \sin^2\theta \tag{11}$$

$$\therefore r_e = \sqrt{r^2 + f^2 \sin^2\theta} \tag{12}$$

In prolate spheroidal co-ordinates, σ is the co-ordinate variable corresponding to confocal ellipsoidal shells. In this co-ordinate system, σ has a simple relation to the foci of an ellipse of semi-major axis r_e, namely that the sum of distance from any point on the ellipse to the two foci is a constant, $2f\sigma = d_1 + d_2$, where d_1 and d_2 are foci distances to a point. Through simple trigonometric manipulation one can easily show

that $\sigma = \frac{r_e}{f}$, and hence

$$\sigma = \frac{\sqrt{r^2 + f^2 \sin^2 \theta}}{f}. \tag{13}$$

By similar argument, we can state that

$$\sigma_n = \frac{r_n}{f}. \tag{14}$$

These can be substituted for σ and σ_n into equation 10, and after simplification this yields the spherical co-ordinate form presented in the main text. If preferred, an alternative form specified on the exterior boundary is also possible, given by

$$P_P(r,\theta) = p_o + \frac{a\Omega}{6D}\left(r^2 - r_o^2 + f^2 \sin^2\theta + f^2 \log\left(\frac{r^2 - f^2\cos^2\theta}{r_o^2 - f^2}\right) + \left(\frac{r_n^3}{f}\right) \log\left(\frac{(f + \sqrt{r^2 + f^2\sin^2\theta})(f - r_o)}{(f - \sqrt{r^2 + f^2\sin^2\theta})(f + r_o)}\right)\right). \tag{15}$$

Oblate spheroids

We solve for the oblate case in oblate spherical coordinates [4], and employ similar methods to the prolate case. Assuming azimuthal symmetry, the Laplacian in this coordinate system is

$$\nabla^2 P = \frac{1}{a^2\left(\sigma^2 - \tau^2\right)} \left\{ \frac{\sqrt{\sigma^2 - 1}}{\sigma} \frac{\partial}{\partial \sigma} \left[\left(\sigma \sqrt{\sigma^2 - 1} \right) \frac{\partial P}{\partial \sigma} \right] + \frac{\sqrt{1 - \tau^2}}{\tau} \frac{\partial}{\partial \tau} \left[\left(\tau \sqrt{1 - \tau^2} \right) \frac{\partial P}{\partial \tau} \right] \right\}. \tag{16}$$

An approach analogous to the prolate case can be taken, with the same boundary conditions. Again all τ terms cancel, yielding

$$P_O(\sigma) = \frac{a\Omega f^2}{6D}\left(\sigma^2 - \sigma_n^2 + 4\log\left(\frac{\sigma}{\sigma_n}\right) + 2(\sigma_n^2 + 2)(\sqrt{\sigma_n^2 - 1})\left(\arctan\left(\frac{1}{\sqrt{\sigma^2 - 1}}\right) - \arctan\left(\frac{1}{\sqrt{\sigma_n^2 - 1}}\right)\right)\right) \tag{17}$$

which as in the prolate case satisfies the boundary conditions and is a solution to the reaction-diffusion equation to be solved. Following the same conversion to spherical co-ordinates as described in the prolate case yields the equation in the main text.Alternatively, one may prefer an alternative definition of

$$P_O(r,\theta) = p_o + \frac{a\Omega}{6D}\left(r^2 - r_o^2 + f^2\sin^2\theta + 2f^2\log\left(\frac{r^2 + f^2\sin^2\theta}{r_o^2}\right) + \frac{2(r_n^2 + 2f^2)(\sqrt{r_n^2 - f^2})}{f}\left(\arctan\left(\frac{f}{\sqrt{r^2 - f^2\cos^2\theta}}\right) - \arctan\left(\frac{f}{\sqrt{r_o^2 - f^2}}\right)\right)\right). \tag{18}$$

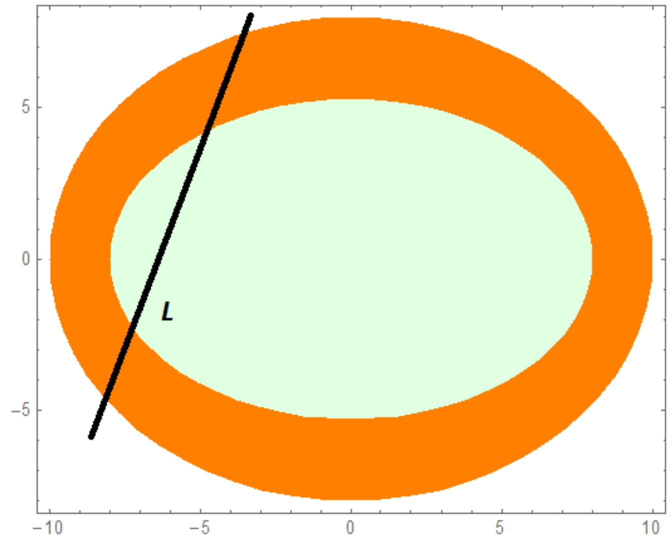

Fig 2. A plane cut (represented in 2D as a line L) going through confocal ellipsoidal spheroids. The z-axis is along the horizontal, and x-axis along vertical. This does not pass through the ellipsoid common centers.

Proof: Planes through an ellipsoid can only produce confocal inner/ outer ellipses if the plane passes through the common centre with slope $m = 0$.

Any plane through an ellipsoid produces an ellipse [5]. Here we will show that for two confocal enclosed ellipsoids, a cut that does not go through their common centre cannot produce confocal ellipses. Consider the two dimensional projection of an ellipsoid in figure 2 for simplicity. The plane is represented by the line L of slope m, which goes through a point $(z_o, 0)$ where $z_o \neq 0$.The equation of this line is given by

$$x = m(z - z_o) \tag{19}$$

and the equations of the outer ellipse (semi-major axis a_o semi-minor axis b_o), and inner ellipse (semi-major axis a_i semi-minor axis b_i) projections are given by

$$\frac{z^2}{a_o^2} + \frac{x^2}{b_o^2} = 1 \tag{20}$$

$$\frac{z^2}{a_i^2} + \frac{x^2}{b_i^2} = 1. \tag{21}$$

L intersects the outer ellipse at two points, which can be found by substituting equation 8 into equation 9 and solving the quadratic expression. The midpoint of these two points is given by

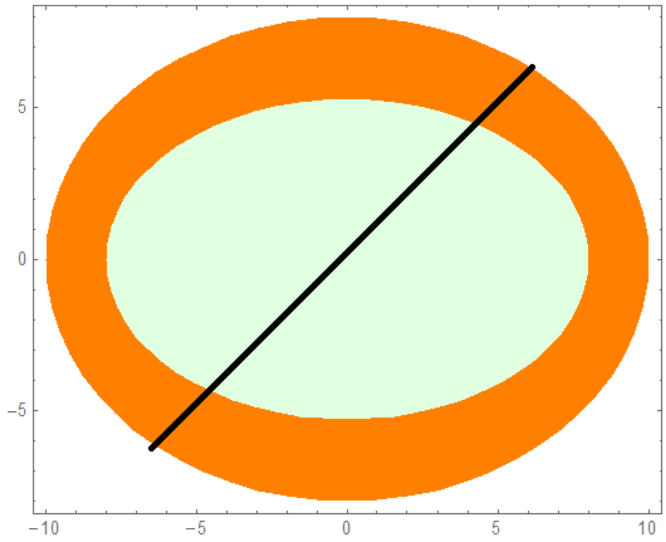

Fig 3. A plane cut (represented in 2D as a line L) going through confocal ellipsoidal spheroids. The z-axis is along the horizontal, and x-axis along vertical. L passes through the common center of the ellipsoids.

$$(z_{o_{Mid}}, x_{o_{Mid}}) = \left(\frac{m^2 z_o}{\frac{b_o^2}{a_o^2} + m^2}, m z_o \left(\frac{m^2}{\frac{b_o^2}{a_o^2} + m^2} - 1 \right) \right) \tag{22}$$

and similarly, the midpoint of the intersections on the inner ellipsoid are given by

$$(z_{i_{Mid}}, x_{i_{Mid}}) = \left(\frac{m^2 z_o}{\frac{b_i^2}{a_i^2} + m^2}, m z_o \left(\frac{m^2}{\frac{b_i^2}{a_i^2} + m^2} - 1 \right) \right). \tag{23}$$

If $z_o \neq 0$, then for these points to coincide, both conditions $z_{i_{Mid}} = z_{o_{Mid}}$ and $x_{i_{Mid}} = x_{o_{Mid}}$ must be satisfied. Taking the latter condition, we can re-arrange and rewrite this condition as being equivalent to

$$\frac{b_o}{a_o} = \frac{b_i}{a_i}. \tag{24}$$

We can re-write a_o in terms of a_i by some scaling factor s_1 and b_o in terms of b_i by some scaling factor s_2, and apply it to the above equation. After re-arrangement, this can be shown to hold if and only if $s_1 = s_2$. But by definition, were this true then both the inner and outer ellipses would have the same eccentricity, e. And if this were true, then

$$e a_o \neq e a_i \tag{25}$$

and thus the resultant ellipses cannot ever be confocal, either having differing centers

$(z_{o_{Mid}}, x_{o_{Mid}}) \neq (z_{i_{Mid}}, x_{i_{Mid}})$ or being non-confocal even if they share a center. A similar argument can be applied even for planes through the center, as illustrated in figure 3. In this instance, the inner and outer ellipsoids share a common center at the origin. As $z_o = 0$, we can establish the intersection points between the line and the outer ellipsoid, and show the semi-major axis length is given by

$$a_{o_s} = \sqrt{\frac{m^2+1}{\frac{1}{a_o^2} + \frac{m^2}{b_o^2}}} \tag{26}$$

where the other axis length by symmetry arguments for spheroids is simply b_o. A similar argument gives the axis length for the inner ellipsoid cut by

$$a_{i_s} = \sqrt{\frac{m^2+1}{\frac{1}{a_i^2} + \frac{m^2}{b_i^2}}}. \tag{27}$$

From the condition of confocality, we can state that

$$\sqrt{a_{o_s}^2 - b_o^2} = \sqrt{a_{i_s}^2 - b_i^2} \tag{28}$$

which becomes after re-arrangement

$$\frac{m^2+1}{\frac{1}{a_o^2} + \frac{m^2}{b_o^2}} - b_o^2 = \frac{m^2+1}{\frac{1}{a_i^2} + \frac{m^2}{b_i^2}} - b_i^2. \tag{29}$$

From inspection, it can be shown that the only value of m that will satisfy the restrictions on this equation is $m = 0$, which is a cut through the centre along the semi-major axis of the ellipsoid. Thus, we can conclude that if the outer and inner ellipses are confocal, the plane section has crossed through the centre and is alone the ellipsoid semi-major axis.

Error analysis / Variance formula

In the main text, the error from equation 15 can be explicitly generated by the variance formula shown in equation 16. After the requisite calculus, this would yield

$$\Delta a\Omega_W = \frac{12Dp_o}{(r_o - r_n)^3(r_o + 2r_n)^2}\sqrt{(r_o^2 + r_o r_n + r_n^2)^2(\Delta r_o)^2 - 9(r_o r_n)^2(\Delta r_n)^2}. \quad (30)$$

This is the uncertainty estimate for a spherical assumption of ellipsoidal OCR. It is encoded in the Mathematica files in **S2** alongside the other error metrics discussed in the paper.

References

1. Prolate spheroidal coordinates (Wikipedia entry); 2018. Available from: `https://en.wikipedia.org/wiki/Prolate_spheroidal_coordinates`.
2. Grimes DR, Kelly C, Bloch K, Partridge M. A method for estimating the oxygen consumption rate in multicellular tumour spheroids. Journal of the Royal Society, Interface / the Royal Society. 2014;11(92):20131124. doi:10.1098/rsif.2013.1124.
3. Grimes DR, Fletcher AG, Partridge M. Oxygen consumption dynamics in steady-state tumour models. Royal Society open science. 2014;1(1):140080.
4. Oblate spheroidal coordinates (Wikipedia entry); 2018. Available from: `https://en.wikipedia.org/wiki/Oblate_spheroidal_coordinates`.
5. Ferguson CC. Intersections of ellipsoids and planes of arbitrary orientation and position. Journal of the International Association for Mathematical Geology. 1979;11(3):329–336.
